# Azoxymethane-Induced Colorectal Cancer Mice Treated with a Polyphenol-Rich Apple Extract Show Less Neoplastic Lesions and Signs of Cachexia

**DOI:** 10.3390/foods10040863

**Published:** 2021-04-15

**Authors:** Florencio Marzo, Fermin I. Milagro, Jaione Barrenetxe, María Teresa Díaz, J. Alfredo Martínez

**Affiliations:** 1 Laboratory of Animal Physiology and Nutrition, School of Agronomy, Universidad Pública de Navarra, 31006 Pamplona, Spain; marzo@unavarra.es; 2 ISFOOD (Research Institute for Innovation & Sustainable Development in Food Chain), Universidad Pública de Navarra, 31006 Pamplona, Spain; 3 Center for Nutrition Research, Department of Nutrition, Food Science and Physiology, University of Navarra, 31008 Pamplona, Spain; jaiobar@unav.es (J.B.); jalfmtz@unav.es (J.A.M.); 4 Navarra Institute for Health Research (IdISNA), 31006 Pamplona, Spain; 5 Centro de Investigación Biomédica en Red de la Fisiopatología de la Obesidad y Nutrición (CIBERobn), Instituto de Salud Carlos III, 28029 Madrid, Spain; 6 Departamento de Tecnología de Alimentos, Instituto Nacional de Investigación Agraria (INIA), Carretera de la Coruña, km7, 28040 Madrid, Spain; diaz.teresa@inia.es

**Keywords:** obesity, polyps, aberrant crypt foci, chlorogenic acid, phloridzin

## Abstract

Obesity is considered a risk factor for the development of colorectal cancer. In rodents, high-fat (HF) diets are able to increase the formation of azoxymethane (AOM)-induced polyps. Polyphenol-rich apple extracts have antioxidant and anti-inflammatory activities and may induce an amelioration of the manifestations of colorectal cancer. Twenty-seven male Crl:CD-1 mice received AOM during four weeks and were subsequently divided into three groups fed a HF diet (*n* = 9 each group): a non-supplemented group, a second group supplemented with apple extract at 1%, and a third group supplemented with the same apple extract at 1.5%. Energy metabolism and the respiratory quotient were not affected by the supplementation with the apple extract. Although body weight was not affected by the treatment, the mice supplemented with the apple extract showed less signs of cachexia than the non-treated mice. In the intestine, the mice supplemented with the apple extract showed lower sucrase, dipeptidyl-peptidase IV, and aminopeptidase N activities, and less intestinal lesions (aberrant crypt foci and polyps). Administration of a polyphenol-rich apple extract reduces the number of neoplastic lesions in mice with AOM-induced colorectal cancer and contributes to preserve adipose tissue mass.

## 1. Introduction

Colorectal cancer is the second most frequent cause of cancer deaths (irrespective of sex) and represents approximately 10% of all cancers. However, it is also one of the most preventable cancers as it has been related with several modifiable risk factors, such as overweight and obesity, sedentarism, smoking, eating red and processed meat, and drinking alcohol, and some protective dietary factors, such as fiber-rich plant foods [1].

Azoxymethane (AOM) intraperitoneal administration is, together with the Apc(Min/+) mouse, the main animal model used to study the effects of chemopreventive agents on colorectal cancer [2]. In rodents, AOM induces colonic mucosal damage and neoplasia, particularly the occurrence of aberrant crypt foci (ACF) [3,4]. ACF are precursors of colon cancer and are being used as a surrogate end point for tumors in animals and humans. ACF are two to three times larger than normal crypts, are microscopically elevated, have a slit-like opening and a thick epithelial lining that stains darker than normal crypts with a large pericryptal zone [5]. In this sense, they provide a simple and economical tool for preliminary screening of potential chemopreventive agents.

A high-fat (HF) diet is usually considered a risk factor in the development of colorectal cancer in animals [6,7]. HF has been associated with an increase in the number and size of large polyps, but not total polyps, in animal models [8]. However, for the moment, little support for an association between dietary fat and colorectal cancer risk has been found in humans [9]. On the other hand, plant foods are rich in bioactive compounds with beneficial effects on disease prevention, including cancer [10,11]. The consumption of freeze-dried vegetables or some plant extracts rich in polyphenols has been reported to reduce the number of aberrant crypt multiplicity and the inhibition of tumor growth in several types of cancer [12,13,14,15]. Some of the phenolic compounds that could likely act as preventive agents against colorectal cancer are quercetin, genistein, curcumin, and epigallocatechin gallate (EGCG) [16,17]. In this sense, apple extracts (AS) and their polyphenols have shown beneficial antioxidant and anti-inflammatory effects in different experimental studies [18,19,20,21]. Moreover, several case-control studies have shown that the adjusted risk of colorectal cancer was inversely correlated with the daily number of apple servings [22,23]. In relation to colorectal cancer models, a crude apple extract from waste, rich in phenolic compounds, beneficially influenced key stages of carcinogenesis in colon cells in vitro, protecting against DNA damage, improving barrier function, and inhibiting invasion [24]. An apple peel extract with powerful antioxidant activity and a mixture of different phenolic compounds extracted from apple, mainly quercetin, epicachetin, chlorogenic acid, phloridzin, and caffeic acid, decreased cell viability and induced apoptosis in in vivo cancer cells [21,25]. In Apc(Min/+) mice, apple polyphenols reduced polyp number and growth, eliminated high-grade dysplasia, and prevented cachexia [25]. In 1,2-dimethylhydrazine (DMH)-induced colon carcinogenesis in rats, dietary supplementation with polyphenol-rich apples reduced inflammation and increased apoptosis. However, all the animal studies have been performed with diets with low or moderate amount of fat [21,25].

Given that previous studies have suggested the potential beneficial effects of AS on the reduction of colorectal cancer occurrence, the objective of the present study was to evaluate the effects of an apple extract of known composition on the metabolic complications of AOM-induced colorectal cancer in mice fed a HF diet.

## 2. Materials and Methods

### 2.1. Analysis of Phenolic Compounds by HPLC

The powdered apple extract was dissolved in a water:methanol solution (50:50 *v*/*v*) at a concentration of 2.5 mg Ml^−1^ After filtration, the extracts were injected with a 45 μm syringe filter directly into HPLC system. The HPLC system was composed of a Waters 2695 instrument and a variable detector VIS/UV Waters 996 (Waters, Milford, MA, USA). Separation was achieved at 40 °C using a column LiChrospher RP-18 (250 × 4 mm and 5 µm, Merck, Darmstadt, Germany) protected by a precolumn LiChrospher 100 RP-18 (30 × 4 mm and 5 µm (Merck). The injections for extract samples and standards were 20 μL. The solvent system for dihydrochalcone, flavonols, and polyphenolics acids was composed of methanol (solvent A) and 2% acetic acid in water (solvent B). Elution was performed at 0.8 mL min^−1^ with the following gradient program of solvent A: 0–50 min, 0–100%; 50–60 min, 100%; 60–65 min, 100–0%; 65–80 min, 0%. The solvent system for flavanols was methanol (solvent A) and 0.06% perchloric acid in water (solvent B). Elution was performed at 1 mL min^−1^ with the following gradient program of solvent A: 0–55 min, 0–22%; 55–65 min, 22–100%; 65–75 min, 100%; 75–100 min, 100–0%. Dihydrochalcones and flavanols were detected at 280 nm, polyphenolic acids at 320 nm, and flavonols at 360 nm. Calibration curves of pure standards were used for quantification.

### 2.2. Animals and Treatments

Twenty-seven male Crl:CD-1 mice (ICR, Charles River Laboratories Inc., Barcelona, Spain) were used, with an initial body weight of 26.8 ± 1.2 g. The animals were housed in filtered-cages containing nesting material under controlled conditions (22 ± 2 °C of temperature and 55% ± 5% of humidity), under a 12:12-h light-dark cycle. All the animals received a high-fat diet (Purified Diet 235HF; SAFE, Barcelona, Spain) with 46.0% of energy as fat. The composition of the experimental diets is shown in Table 1.

After one week of acclimatization, all the animals received once a week, during four weeks, an intraperitoneal dose of 10 mg AOM/kg body weight. Then, the mice were divided into three groups (*n* = 9 each group): an AOM group fed the HF diet, and two AOM groups fed the same HF diet supplemented with an apple extract (SelectSieve^®^ Apple PCQ, Principium SA, Viganello, Switzerland) at 1% (AS1), and 1.5% (AS15), respectively.. The experimental period lasted 13 weeks, during which animals had *ad libitum* access to food and water. At the endpoint, the animals showed no sign of rectal bleeding. Five additional Crl:CD-1 mice were fed the HF diet but did not receive AOM and were used as healthy controls.

Body weight and the consumption of food and water were recorded daily. At the end of the study, all animals were anesthetized with CO_2_ and killed by guillotine, and organs were dissected, weighed and stored in liquid nitrogen. The study was approved by the Ethics Committee of the Universidad Pública de Navarra (approval code PI:07/06). and the sacrifice followed the procedures established in the guide of the Canadian Council of Animal Care [26].

### 2.3. Indirect Calorimetry

In the 6th and 12th weeks, indirect calorimetry measurements were performed. The animals were housed in metabolic cages (Oxylet, Panlab SL, Barcelona, Spain) and oxygen consumption (VO_2_) and CO_2_ production (VCO_2_) were recorded at 3 min intervals for 2 h. Respiratory quotient (RQ) was calculated as the ratio of VCO_2_ to VO_2_. Total energy expenditure (TEE) was calculated with the Weir equation [27], modified according to García-Diaz et al [28] in order to refer the data to the metabolically active weight:

TEE (kcal/day/kg body weight^0.75^) = VO_2_ (mL/min) × 1.44 × [3.815 + [1.232 × RQ]]

### 2.4. Enzyme Activities

Brush-border membrane vesicles were prepared according to the procedure described by Shirazi-Beechey et al. [29]. Protein content of the brush-border membrane vesicles was determined by the Bradford method [30]. The activities of the enzymes sucrase, maltase, aminopeptidase N, and dipeptidyl peptidase-4 were analyzed as previously described [31]. 

### 2.5. Histopathological Assessment

The rodent model of AOM-induced colorectal cancer has been widely used to evaluate anticarcinogenic properties of dietary factors as azoxymethane induces the growth of putative premalignant lesions called ACF [32].

The animals were euthanized at the end of treatment and the colons were removed. Each colon was flushed with physiologic saline, and cut open longitudinally along the mesenteric attachment.

For identification of colonic ACF, the formalin-fixed (10% buffered formalin overnight) colonic tissues were cut into middle (3–4 cm from anus) and distal (1–3 cm from anus) segments, and then transferred to 70% ethanol. The distal segment was stained in a 0.2% methylene blue solution for 10 min. ACF were identified under a microscope (40×) as large thick crypts that are more darkly stained than normal crypts (Supplementary Figure S1). The number of ACF observed per distal colon was recorded. ACF containing more than 3 AC/focus were classified as ACF in this study. The lesions with crypt multiplicity were considered large ACF by light microscopy, which were also defined elsewhere as tumors. ACF were evaluated using the method described by Bird [33] and classified by the method of Paulsen et al. [34]

The middle segments of the colon were evaluated histopathologically with blinded review. A 0.5-cm section was taken from the most distal region of the middle segments of colon. The colon tissue was processed and embedded in paraffin and were cut (5 µm) serially for at least 10 slides and stained with hematoxylin and eosin. Tissue slides were examined in a light microscope.

### 2.6. Fatty Acid Analysis

Fatty acid methyl esters (FAMES) of the feed and feces were performed in duplicate, following the method described by Sukhija and Palmquist [35]. FAMES of freeze-dried liver were formed in duplicate, according to the method proposed by Lee et al. [36]. The final organic layers with the FAMES were transferred to a 2 mL vial and stored at −20 °C until analysis.

FAMES from feed, feces and liver were analyzed by gas chromatography equipped with a flame ionization detector (Perkin-Elmer Autosystem-1:A, Massachusetts, USA) and an Omegawax™ 320 capillary column (30 m × 0.32 mm i.d., 0.25 μm film thickness) (Supelco, Bellefonte, PA, USA). Samples were injected (1.0 μL) in the split mode at a 1:50 split ratio with helium as the carrier gas at a constant flow of 9 psig. The detector and injector oven temperatures were set at 255 °C and 250 °C, respectively. The temperature profile of the oven was 150 °C for 1 min, increasing by 6 °C/min to 190 °C and after that, 1 °C/min to 210 °C and held for 23 min. The different FAMES were identified by comparison with their standard (FAME mixture; Sigma-Aldrich, St. Louis, MO, USA). Results were expressed as a percentage of the total FAMES.

### 2.7. Statistical Analysis

Results are expressed as mean ± standard deviation (SD). Differences among the experimental groups were analyzed by one-way ANOVA and Duncan’s post-hoc test. Histological data were analyzed by Kruskal-Wallis test followed by Mann-Whitney U-test. A *p* value < 0.05 was considered statistically significant for all results. SPSS Statistics 21.0 software (IBM Corp., New York, NY, USA) was used for statistical analyses.

## 3. Results

Phenolic compounds present in the apple extract and their concentrations are listed in Table 2. Chlorogenic acid (CGA) was present in the highest concentration, followed by phloridizin, phloretin, and epicatechin. Quercetin was present at low concentrations. It has been estimated that a 200 mL cup of roast and ground coffee might supply from 20 to 675 mg CGA depending on type of roast [37]. Thus, the dose of CGA used in this study corresponds to the average intake consumed by human while drinking three cups of coffee per day.

Food intake at the beginning of the experimental period was 0.22 ± 0.03 (grams of food per gram of body weight). At the end of the experimental period, all groups decreased food intake (35–40%), with no statistical differences between groups (data not shown). Body weight gain was similar in the different groups (Table 3), but the amount of perirenal fat was higher in the groups treated with AS. No statistical differences were observed between groups in the weight of the other organs and fat depots (Table 3).

Energy metabolism was not affected by the supplementation with the AS. The three groups showed a RQ ranged from 0.82 ± 0.06 to 0.84 ± 0.06. No significant differences were detected in TEE. At 12th week were 271.4 ± 52.0 (HF), 272.0 ± 52.9 (AS1), and 263.9 ± 51.2 (AS1.5) kcal/day/kg body weight0.75.

Mice supplemented with AS showed reduced sucrase activity in a dose-dependent manner (Table 4). However, maltase activity was not affected by the supplementation. Both, aminopeptidase N and dipeptidyl peptidase-4 activities were reduced in the AS1.5 group, although the dipeptidyl peptidase-4 activity was slightly increased in the AS1 group.

**Figure 1:**
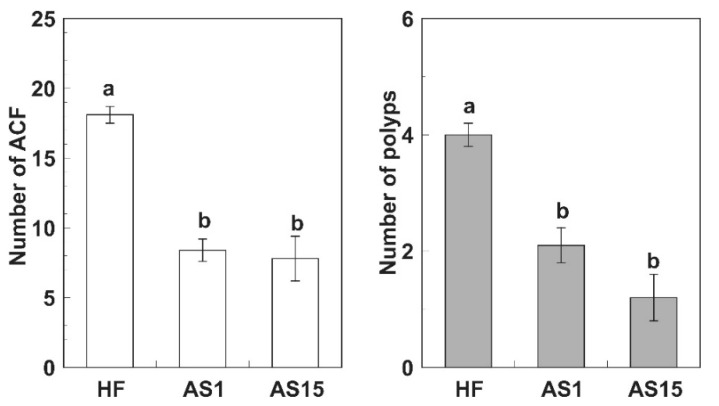
Number of aberrant crypt foci (ACF) and number of polyps in the three experimental groups: high-fat diet (AOM-HF); HF supplemented with apple extract 1% (AOM-HF-AS1); and HF supplemented with apple extract 1.5% (AOM-HF-AS1.5). Data are Mean ± SEM. Different letters are significant at *p* < 0.05 by Mann-Whitney U-test.

The data about density, number and anatomical location of ACF are based on the study of colorectal resections. It was found that ACF increased from the proximal to the distal region of the colon. Non-supplemented mice had a higher average number of ACF and tumors in the distal colon than those supplemented with AS, which demonstrates that the administration of the polyphenol-rich AS significantly reduced the pathological alterations of the colon. The inhibitory effect of AS on ACF was not dose-dependent. The crypt multiplicity (number of aberrant crypts/focus) did not differ between both doses of the polyphenol-rich AS. Neither ACF nor tumors were detected in the AOM-untreated (healthy control) mice; they were only observed in the three groups of AOM-administered animals (Figure 1). 

The histological evaluation showed that the AOM-administered mice had hyperplastic and dysplastic crypts. The tissue architecture alterations comprise crypt multiplicity [32], mucosa alteration [38], dysplasia [39], mucin depletion [40], immune cell infiltration [41], and hyperchromasia with mitotic activity [42]. In the present study, the administration of AS significantly reduced the effect of AOM in the middle segments of the colon, as observed by microscope.

In feces and urine, the most abundant phenolic compounds were chlorogenic acid, caffeic acid, and phloridzin (Table 5). All the detected compounds were found in a dose-dependent manner, being higher in the AS1.5 group.

The most abundant fatty acids in the liver were the monounsaturated fatty acid (MUFA) C:18:1 (oleic acid) and the saturated fatty acid (SFA) C16:0 (palmitic acid), while in the feces the major fatty acids were the SFA C18:0 (estearic acid) and C16:0 for all treatments (Table 6). Polyunsaturated fatty acids (PUFA) percentages, such as those of C18:2 (linoleic acid) or C20:4 (arachidonic acid), were low in faeces, but higher percentages were observed in the liver (14.8% and 6.89% for C18:2 and C20:4 respectively). Fatty acid composition of both liver and feces was not affected by AS supplementation.

## 4. Discussion

Chemoprevention is defined as the use of natural compounds that can delay, prevent, or even reverse the development of adenomas, as well as the progression from adenoma to carcinoma. The molecular mechanisms of their chemopreventive action are associated with the modulation of signaling cascades and the expression of genes involved in the regulation of cell proliferation, differentiation, and apoptosis and the suppression of chronic inflammation, metastasis, and angiogenesis [43]. Numerous compounds occurring in plant foods exert their anticarcinogenic effects through either blocking cancer initiation or suppressing promotion [44]. In this context, anticarcinogenic properties have been attributed to some polyphenol-rich extracts. As there are no animal models that develop colon cancer spontaneously, AOM is commonly used to induce tumors, which are histologically similar to those found in humans [4].

Cachexia is one of the signs usually accompanying cancer and is associated with a poor prognosis. In the present study, the administration of AS contributed to protect the loss of perirenal fat depots, although this protective effect was not observed in other fat depots. This result is corroborated by RQ values of around 0.82–0.84 in all groups, suggesting the use of proteins as a primary fuel source, but it also suggests fat mobilization from the adipose tissue and dietary fat oxidation [45]. The present study shows that AS supplementation does not affect the TEE. Other studies showed contradictory effects. A transient increase in energy expenditure at treatment initiation was observed in HF-fed mice supplemented with 0.8% quercetin [46], while a decrease in energy expenditure was appreciated in HF-fed animals supplemented with 0.5–1% EGCG [47].

The results of gut enzyme activities are consistent with those obtained by other authors when quercetin was included in the diet of healthy animals [31]. On the other hand, the intestinal disaccharidase activity in diabetic rats was observed to be reduced by a quercetin-supplemented diet [48]. The lower nitrogen uptake in the groups supplemented with AS is probably associated with increased protein catabolism. In this context, an increased fecal N excretion was observed in healthy rats whose diet was supplemented with tea extract. It has been reported that polyphenols can bind to dietary protein and decrease absorption of dietary nitrogen. In fact, it has already been described that endogenous nitrogen may make a significant contribution to the observed increase in fecal N [49]. Our data suggest that, at the doses tested, the effects of the polyphenol-rich extract are not dose-dependent for most of the parameters measured. However, according to the data shown in Table 4 regarding the disaccharidase and peptidase enzyme activities in the jejunum, a dose-dependent effect is observed. This could be explained because these enzymes are located on the surface of the colonocytes, and the polyphenols can act more directly over them.

Previous studies have shown that HF diets are associated with an increase in the number of large polyps [50,51], and with the development of ACF by activation of β-catenin [52]. The present study confirms the inhibitory effect of a polyphenol-rich AS on mouse colon tumorigenesis model fed a HF diet. Previous studies providing a diet supplemented with apples have shown a reduction of intestinal lesions, specifically of the number of polyps [25] and ACF [53], in mice with colon cancer. Certain polyphenols, such as chlorogenic acid, have been reported to decrease the multiplicity of AOM-induced colon tumors in rats [54]. Others, like EGCG, have been demonstrated to inhibit DNA methyltransferases in vitro. For example, oral administration of EGCG showed a dose-dependent effect on the levels of intestinal S-adenosylmethionine and reduced DNA methylation in mice [55]. Also, a reduction of ACF was observed in AOM-induced colon cancer rats fed a HF diet when they received a preparation consisting of 65% EGCG and 22% of other catechins. In these rats, increased apoptosis and decreased β-catenin levels were also observed [56]. In ApcMin/+ mice treated with the same preparation, apoptosis stimulation and a decrease in β-catenin expression were observed [57].

At the genetic level, mutations in genes implicated in the Wnt signaling pathway occur in crypt formation. At the subcellular level (membrane, cytoplasm and nucleus compartments), alterations of signaling proteins (β-catenin and E-cadherin) occur in the regulation of crypt production and maintenance [58]. Polyphenols like EGCG have been reported to inhibit the Wnt/β-catenin pathway, which is aberrantly upregulated in colorectal cancers. For example, EGCG suppresses the β-catenin response transcription activated by Wnt3a-conditioned medium [59]. 

In addition to Wnt/β-catenin, EGCG has shown to reduce the level of cyclooxygenase (COX)-2, one of the main mediators in the inflammatory signaling pathway, by inhibiting the activation of the epidermal growth factor receptor (EGFR) in CRC cells [60]. The ability of EGCG to bind the tyrosine domain of EGFR inhibiting its activation is widely known [60], as is the fact that 60–80% of cases of sporadic colorectal cancer (CRC) are associated with EGFR expression [61]. 

The deposition of fatty acids in the liver depends on dietary fatty acids and de novo lipogenesis [62]. This is consistent with data observed in the present study, with high content of C16:0 and C18:0 in the mice diets and liver. C18:0 serves as a substrate for endogenous synthesis of C18:1 [63], which can explain the high proportion of C18:1 observed in the liver. The fatty acid composition of feces can be affected by dietary fatty acids, intestinal fatty acid absorption, and by the activity of colonic bacteria [64]. In this study, fatty acid composition of the feces reflected the fatty acid composition of the diets, and the supplementation did not significantly affect fatty acid composition of liver and feces.

## 5. Conclusions

The present work demonstrates the inhibitory effects of a polyphenol-rich apple extract on the development of polyps and ACF in AOM-treated mice fed a HF diet.

## Figures and Tables

**Table 1:** Composition of the high-fat (HF) experimental diet.

Component	Content (g kg^−1^ Diet)
Casein *	200.0
Cornstarch *	130.0
Sucrose *	293.0
Cellulose *	50.0
Maltodrextrin *	22.0
Lard *	200.0
Soy oil *	25.0
Mineral additive (SAFE mineral 205B) *	70.0
Vitamin additive (SAFE vitamin 200) *	10.0
Fatty acid profile **	
Lauric acid (C12:0)	27.4
Myristic acid (C14:0)	5.9
Pentadecanoic acid (C15:0)	0.6
Palmitic acid (C16:0)	107.0
Heptadecanoic acid (C17:0)	1.4
Stearic acid (C18:0)	56.7
Palmitoleic acid (C16:1)	1.4
Oleic cid (C18:1)	22.9
Linoleic cid (C18:2)	1.4
α-Linolenic acid (C18:3)	0.3

* Data according to the manufacturer. ** Data according analysis.

**Table 2:** Polyphenol compounds of the apple extract, assessed by high-performance liquid chromatography.

Compound	Content (g kg^−1^)
*Phenolic acids*	
Chlorogenic acid	165.3 ± 2.4
p-Coumaric acid	30.7 ± 1.7
Caffeic acid	58.7 ± 20.0
Ferulic acid	49.2 ± 2.4
Gallic acid	nd
*Dihydrochalcones*	
Phloridzin	122.2 ± 3.5
Phloretin	4.5 ± 0.1
Phloretin derivative	38.7 ± 1.1
*Flavanols*	
Epicatechin	35.9 ± 1.3
Catechin	14.2 ± 0.2
Procyanidin B1	13.8 ± 0.2
Procyanidin B2	25.2 ± 0.5
*Flavonols*	
Quercetin	4.9 ± 0.3
Quercetin-3-glucoside	2.8 ± 0.2

Values are mean ± SD (*n* = 4).

**Table 3:** Body and organ weights (in grams) in the three experimental groups.

	AOM-HF	AOM-HF- AS1	AOM-HF- AS1.5
Initial body weight (week 0)	26.8 ± 1.3	26.8 ± 1.2	26.8 ± 1.2
Final body weight (week 12)	40.0 ± 4.5	38.9 ± 2.3	39.0 ± 3.4
Liver	1.454 ± 0.229	1.384 ± 0.191	1.483 ± 0.152
Spleen	0.145 ± 0.071	0.135 ± 0.079	0.155 ± 0.075
Kidneys	0.562 ± 0.057	0.525 ± 0.079	0.542 ± 0.047
Gastrocnemius muscle	0.187 ± 0.024	0.187 ± 0.021	0.194 ± 0.024
Jejunum	0.311 ± 0.040	0.314 ± 0.043	0.318 ± 0.044
Colon	0.191 ± 0.050	0.190 ± 0.043	0.179 ± 0.028
Epididimal fat depot	0.943 ± 0.449	1.111 ± 0.414	0.994 ± 0.472
Suprarenal fat depot	0.217 ± 0.090 ^b^	0.313 ± 0.093 ^a^	0.314 ± 0.086 ^a^
Abdominal fat	0.294 ± 0.092	0.365 ± 0.113	0.296 ± 0.170

Values are mean ± SD (*n* = 9 mice/group). Different superscripts in the same row denote significant differences at *p* < 0.05 as assessed by the Duncan’s multiple range test. AOM-HF, high-fat diet; AOM-HF-AS1, high-fat diet + apple extract 1%; AOM-HF-AS1.5, high-fat diet + apple extract 1.5%.

**Table 4:** Disaccharidase and peptidase enzyme activities in the jejunum of the three experimental groups.

Enzyme Activity (nmol Substrate mg^−1^ Protein min^−1^)	AOM-HF	AOM-HF-AS1	AOM-HF-AS1.5
Sucrase	1082 ± 102 ^a^	856 ± 91 ^b^	758 ± 79 ^c^
Maltase	1089 ± 74	1077 ± 96	1040 ± 62
Dipeptidyl-peptidase IV	619 ± 28 ^b^	704 ± 42 ^a^	453 ± 17 ^c^
Aminopeptidase N	6091 ± 925 ^a^	5879 ± 937 ^a^	4794 ± 815 ^b^

Values are mean ± SD (*n* = 9 mice/group). Different superscripts in the same row denote significant differences at *p* < 0.05 as assessed by the Duncan’s multiple range test. AOM-HF, high-fat diet; AOM-HF-AS1, high-fat diet + apple extract 1%; AOM-HF-AS1.5, high-fat diet + apple extract 1.5%.

**Table 5:** Phenolic compounds excreted (mg/100 g) by faeces and urine in the three experimental groups.

	Faeces			Urine		
Compound	AOM-HF	AOM-HF-AS1	AOM-HF-AS1.5	AOM-HF	AOM-HF-AS1	AOM-HF-AS1.5
Chlorogenic acid	nd	5.18 ± 2.23	9.23 ± 3.53 *	nd	4.64 ± 2.94	5.33 ± 2.21
p-Coumaric acid	nd	2.90 ± 1.71	4.60 ± 3.18	nd	1.81 ± 0.55	3.99 ± 0.21 *
Caffeic acid	nd	4.79 ± 3.66	6.50 ± 3.75	nd	1.81 ± 0.30	3.33 ± 1.06
Ferulic acid	nd	0.57 ± 0.43	1.00 ± 0.81	nd	0.63 ± 0.10	1.22 ± 0.21 *
Gallic acid	nd	nd	nd	nd	nd	nd
Phloridzin	nd	4.05 ± 1.39	5.50 ± 1.82	nd	1.57 ± 0.62	2.92 ± 1.33
Phloretin	nd	0.32 ± 0.25	0.38 ± 0.25	nd	0.30 ± 0.30	1.97 ± 0.45 *
Phloretin derivative	nd	1.55 ± 0.25	2.63 ± 0.97 *	nd	nd	nd
Epicatechin	nd	14.47 ± 8.30	28.39 ± 14.10	nd	nd	nd
Catechin	nd	7.91 ± 3.59	9.62 ± 3.63	nd	nd	nd
Procyanidin B1	nd	3.50 ± 1.25	6.49 ± 2.95 *	nd	nd	nd
Procyanidin B2	nd	1.43 ± 0.99	1.93 ± 0.89	nd	nd	nd
Quercetin	nd	nd	0.26 ± 0.30	nd	0.12 ± 0.01	0.15 ± 0.05
Quercetin-3-glucoside	nd	nd	nd	nd	nd	nd

Values are mean ± SD (*n* = 9 mice/group). Asterisk in the same row denotes significant difference at *p* < 0.05 as assessed by the Student’s test. AOM-HF, high-fat diet; AOM-HF-AS1, high-fat diet + apple extract 1%; AOM-HF-AS1.5, high-fat diet + apple extract 1.5%.

**Table 6:** Fatty acids (mg/100 g total fatty acids) of liver and feces in the three experimental groups.

	Liver			Faeces		
	AOM-HF	AOM-HF-AS1	AOM-HF-AS1.5	AOM-HF	AOM-HF-AS1	AOM-HF-AS1.5
Lauric acid C12:0	nd	nd	nd	0.11 ± 0.03	0,08 ± 0.01	0.07 ± 0.04
Myristic acid C14:0	0.25 ± 0.09	0.31 ± 0.06	0.28 ± 0.11	0.74 ± 0.08	0.71 ± 0.12	0.63 ± 0.07
C15:0	0.07 ± 0.01	0.07 ± 0.01	0.08 ± 0.03	0.37 ± 0.04	0.28 ± 0.12	0.25 ± 0.05
C16:0	20.29 ± 1.89	19.82 ± 1.06	20.02 ± 2.78	20.69 ± 0.35	21.10 ± 0.65	21.54 ±0.13
C17:0	0.25 ± 0.03	0.24 ± 0.03	0.27 ± 0.02	0.66 ± 0.03	0.64 ± 0.04	0.68 ± 0.02
C18:0	9.20 ± 2.47	8.71 ± 2.91	10.63 ± 2.29	46.63 ± 4.31	45.73 ± 4.74	49.28 ± 2.82
C20:0	0.18 ± 0.05	0.19 ± 0.11	0.28 ± 0.11	2.76 ± 0.22	2.54 ± 014	2.45 ± 0.09
C22:0	nd	nd	nd	1.31 ± 0.11	1.21 ± 0.12	1.17 ± 0.10
C23:0	nd	nd	nd	0.25 ± 0.01	0.18 ± 0.04	0.15 ± 0.03
C24:0	nd	nd	nd	0.53 ± 0.05	0.39 ± 0.14	0.50 ± 0.04
C16:1	1.82 ± 0.66	1.99 ± 0.78	1.44 ± 0.56	0.80 ± 0.19	0.78 ± 0.25	0.58 ± 0.14
C17:1	0.18 ± 0.06	0.18 ± 0.04	0.16 ± 0.05	0.11 ± 0.01	0.09 ± 0.01	0.08 ±0.01
C18:1	40.04 ± 8.71	41.76 ± 9.01	38.16 ± 5.12	18.77 ± 2.52	19.64 ± 2.75	17.25 ± 1.84
C20:1n9	0.86 ± 0.30	1.10 ± 0.36	1.23 ± 0.28	1.41 ± 0.36	1.30 ± 0.20	1.19 ± 0.07
C22:1n9	nd	nd	nd	0.40 ±0.09	0.36 ± 0.04	0.37 ± 0.04
C18:2	14.17 ± 1.46	14.84 ± 1.48	15.33 ± 2.00	3.54 ± 0.96	3.98 ± 1.13	2.93 ± 0.79
C18:3	nd	nd	nd	0.40 ± 0.05	0.45 ± 0.09	0.38 ± 0.05
C20:2	nd	nd	nd	0.16 ± 0.02	0.20 ± 0.04	0.20 ± 0.01
C20:3	0.99 ± 0.33	0.92 ± 0.40	1.11 ± 0.29	0.11 ± 0.02	0.11 ± 0.02	0.10 ± 0.03
C20:4	7.38 ± 3.34	6.24 ± 3.74	7.06 ± 2.26	0.24 ± 0.05	0.23 ± 0.02	0.22 ± 0.09
C22:5	0.33 ± 0.13	0.30 ± 0.13	0.32 ± 0.12	nd	nd	nd
C22:6	3.72 ± 1.85	3.14 ± 2.00	3.37 ± 1.15	nd	nd	nd

Values are mean ± SD (*n* = 9 mice/group). AOM-HF, high-fat diet; AOM-HF-AS1, high-fat diet + apple extract 1%; AOM-HF-AS1.5, high-fat diet + apple extract 1.5%.

## Data Availability

The data presented in this study are available on request from the corresponding author.

## Supplementary Materials

The following are available online at https://www.mdpi.com/article/10.3390/foods10040863/s1, Figure S1: Topographical view of ACF. The colons were opened, stained with methylene blue solution and observed on a glass slide by transillumination in an optic microscope (Olympus). Increased size, bright blue staining and flat appearance hidden in the surrounding mucosa were used a criteria to identify ACF (arrow). Original magnification: 10×.

## References

[1] Tárrega López P.J.T., Solera Albero J.S., Rodríguez-Montes J.A. (2014). Primary and secondary prevention of colorectal cancer. Clin. Med. Insights Gastroenterol..

[2] Corpet D.E., Pierre F. (2005). How good are rodent models of carcinogenesis in predicting efficacy in humans? A systematic review and meta-analysis of colon chemoprevention in rats, mice and men. Eur. J. Cancer..

[3] Nandan M.O., Yang V.W. (2010). Genetic and chemical models of colorectal cancer in mice. Curr. Colorectal. Cancer Rep..

[4] Perse M., Cerar A. (2010). Morphological and molecular alterations in 1,2 Dimethylhidrazine and Azoxymethane induced colon carcinogenesis in rats. J. Biochem. Biotech..

[5] Corpet D.E., Tache S. (2002). Most effective colon cancer chemopreventive agents in rats: A systematic review of aberrant crypt foci and tumor data, ranked by potency. Nutr. Cancer.

[6] O´Neill A.M., Burrington C.M., Gillaspie E.A., Lynch D.T., Horsman M.J., Greene M.W. (2016). Higf-fat Western diet-induced obesity contributes to increased tumor growth in mouse models of human colon cancer. Nutr. Res..

[7] Choi S., Snider A.J. (2019). Diet, Lipids and colon cancer. Int. Rev. Cell Mol. Biol..

[8] Chen J., Huang X.F. (2015). High fat diet-induced obesity increases the formation of colon polyps induced by azoxymethane in mice. Ann. Transl. Med..

[9] Lin J., Zhang S.M., Cook N.R., Lee I.-M., Buring J.E. (2004). Dietary fat and fatty acids and risk of colorectal cancer in women. Am. J. Epidemiol..

[10] Matusiewicz M., Baczek K.B., Kosieradzka I., Niemiec T., Grodzik M., Szczepaniak J., Orlińska S., Węglarz Z. (2019). Effect of juice and extracts of saposhnikovia divaricata root on the colon cancer Caco-2 cells. Int. J. Mol. Sci..

[11] Davatgaran-Taghipour Y., Masoomzadeh S., Farzaei M.H., Bahramsoltani R., Karimi-Soureh Z., Rahimi R., Abdollahi M. (2017). Polyphenol nanoformulations for cancer therapy: Experimentale vidence and clinical perspective. Int. J. Nanomed..

[12] Rijken P.J., Timmer W.G., van de Kooij A.J., van Benschop I.M., Wiseman S.A., Meijers M., Tijburg L.B. (1999). Effect of vegetable and carotenoid consumption on aberrant crypt multiplicity, a surrogate end-point marker for colorectal cancer in azoxymethane-induced rats. Carcinogenesis.

[13] Gonzales G.F., Miranda S., Nieto J., Fernández G., Yucra S., Rubio J., Yi P., Gasco M. (2005). Red maca (*Lepidium meyenii*) reduced prostate size in rats. Reprod. Biol. Endocrinol..

[14] Kim H.-A., Jeong K.-S., Kim Y.K. (2008). Soy extract is more potent than genistein on tumor growth inhibition. Anticancer Res..

[15] Niedzwiecki A., Waheed Roomi M., Kalinovsky T., Matthias R. (2016). Aticancer effects of polyphenols and their combinations. Nutrients.

[16] Lambert J.D., Hong J., Yang G., Liao J., Yang C.S. (2005). Inhibition of carcinogenesis by polyphenols: Evidence from laboratory investigations. Am. J. Clin. Nutr..

[17] Gan R.Y., Li H.B., Sui Z.Q., Corke H. (2018). Absorption, metabolism, anti-cancer effect and molecular targets of epigallocatechin gallate (EGCg): An updated review. Crit. Rev. Food Sci. Nutr..

[18] Bellion P., Olk M., Will F., Dietrich H., Baum M., Eisenbrand G., Janzowski C. (2009). Formation of hydrogen peroxide in cell culture media by apple polyphenols and its effect on antioxidant biomarkers in the colon cell line HT-29. Mol. Nutr. Food Res..

[19] Carrasco-Pozo C., Speisky H., Brunser O., Pastene E., Gotteland M. (2011). Apple peel polyphenols protect against gastrointestinal mucosa alterations induced by indomethacin in rats. J. Agric. Food Chem..

[20] D’Argenio G., Mazzone G., Tuccillo C., Ribecco M.T., Graziani G., Gravina A.G., Caserta S., Guido S., Fogliano V., Caporaso N. (2012). Apple polyphenols extract (APE) improves colon damage in a rat model of colitis. Digest. Liver Dis..

[21] Femia A.P., Luceri C., Bianchini F., Salvadori M., Salvianti F., Pinzani P., Dolara P., Calorini L., Caderni G. (2012). Marie Ménard apples with high polyphenol content and a low-fat diet reduce 1,2-dimethylhydrazine-induced colon carcinogenesis in rats: Effects on inflammation and apoptosis. Mol. Nutr. Food Res..

[22] Annema N., Heyworth J.S., McNaughton S.A., Iacopetta B., Fritschi L. (2011). Fruit and vegetable consumption and the risk of proximal colon, distal colon, and rectal cancers in a case-control study in western Australia. J. Am. Diet Assoc..

[23] Jedrychowski W., Maugeri U., Popiela T., Kulig J., Sochacka-Tatara E., Pac A., Sowa A., Musial A. (2010). Case–control study on beneficial effect of regular consumption of apples on colorectal cancer risk in a population with relatively low intake of fruits and vegetables. Eur. J. Cancer Prev..

[24] McCann M.J., Gill C.I.R., O’ Brien G., Rao J.R., McRoberts W.C., Hughes P., McEntee R., Rowland I.R. (2007). Anti-cancer properties of phenolics from apple waste on colon carcinogenesis in vitro. Food Chem. Toxicol..

[25] Fini L., Piazzi G., Daoud Y., Selgrad M., Maegawa S., Garcia M., Fogliano V., Romano M., Graziani G., Vitaglione P. (2011). Chemoprevention of intestinal polyps in ApcMin/+ mice fed with western or balanced diets by drinking annurca apple polyphenol extract. Cancer Prev. Res..

[26] Canadian Council on Animal Care E., Olfert E., Cross B., McWilliam A. (1993). Guide to the Care and Use of Experimental Animals.

[27] Weir J.B. (1949). New methods for calculating metabolic rate with special reference to protein metabolism. J. Physiol..

[28] García-Díaz D., Campion J., Milagro F., Lomba A., Marzo F., Martínez J. (2007). Chronic mild stress induces variations in locomotive behavior and metabolic rates in high fat fed rats. J. Physiol. Biochem..

[29] Shirazi-Beechey S.P., Davies A.G., Tebbutt K., Dyer J., Ellis A., Taylor C.J., Fairclough P., Beechey R.B. (1990). Preparation and properties of brush-border membrane vesicles from human small intestine. Gastroenterology.

[30] Bradford M.M. (1976). A rapid and sensitive method for the quantitation of microgram quantities of protein utilizing the principle of protein-dye binding. Anal. Biochem..

[31] Barrenetxe J., Aranguren P., Grijalba A., Martinez-Peñuela J.M., Marzo F., Urdaneta E. (2006). Effect of dietary quercetin and sphingomyelin on intestinal nutrient absorption and animal growth. Br. J. Nutr..

[32] Bird R.P. (1995). Role of aberrant crypt foci in understanding the pathogenesis of colon cancer. Cancer Lett..

[33] Bird R.P. (1987). Observation and quantification of aberrant crypts in the murine colon treated with a colon carcinogen: Preliminary findings. Cancer Lett..

[34] Paulsen J.E., Løberg E.M., Ølstørn H.B., Knutsen H., Steffensen I.-L., Alexander J. (2005). Flat dysplastic aberrant crypt foci are related to tumorigenesis in the colon of azoxymethane-treated rat. Cancer Res..

[35] Sukhija P.S., Palmquist D.L. (1988). Rapid method for determination of total fatty acid content and composition of feedstuffs and feces. J. Agric. Food Chem..

[36] Lee M.R.F., Tweed J.K.S., Kim E.J., Scollan N.D. (2012). Beef, chicken and lamb fatty acid analysis—A simplified direct bimethylation procedure using freeze-dried material. Meat Sci..

[37] Clifford M.N. (1999). Chlorogenic acids and other cinnamates—Nature, occurrence and dietary burden. J. Sci. Food Agric..

[38] Pretlow T.P., Barrow B.J., Ashton W.S., O’Riordan M.A., Pretlow T.G., Jurcisek J.A., Stellato T.A. (1991). Aberrant crypts: Putative preneoplastic foci in human colonic mucosa. Cancer Res..

[39] Roncucci L., Medline A., Bruce W.R. (1991). Classification of aberrant crypt foci and microadenomas in human colon. Cancer Epidemiol. Biomark. Prev..

[40] Thorup I. (1997). Histomorphological and immunohistochemical characterization of colonic aberrant crypt foci in rats: Relationship to growth factor expression. Carcinogenesis.

[41] Femia A.P., Dolara P., Luceri C., Salvadori M., Caderni G. (2009). Mucin-depleted foci show strong activation of inflammatory markers in 1,2-dimethylhydrazine-induced carcinogenesis and are promoted by the inflammatory agent sodium dextran sulfate. Int. J. Cancer.

[42] Ashokkumar P., Sudhandiran G. (2008). Protective role of luteolin on the status of lipid peroxidation and antioxidant defense against azoxymethane-induced experimental colon carcinogenesis. Biomed Pharmacother..

[43] Pan M.-H., Lai C.-S., Wu J.-C., Ho C.-T. (2011). Molecular mechanisms for chemoprevention of colorectal cancer by natural dietary compounds. Mol. Nutr. Food Res..

[44] Chikara S., Nagaprashantha L.D., Singhai J., Horne D., Awasthi S., Singhal S.S. (2018). Oxidative stress and dietary phytochemicals; role in cancer chemoprevention and treatment. Cancer Lett..

[45] Ferrannini E. (1988). The theoretical bases of indirect calorimetry: A review. Metabolism.

[46] Stewart L.K., Soileau J.L., Ribnicky D., Wang Z.Q., Raskin I., Poulev A., Majewski M., Cefalu W.T., Gettys T.W. (2008). Quercetin transiently increases energy expenditure but persistently decreases circulating markers of inflammation in C57BL/6J mice fed a high-fat diet. Metabolism.

[47] Klaus S., Pultz S., Thone-Reineke C., Wolfram S. (2005). Epigallocatechin gallate attenuates diet-induced obesity in mice by decreasing energy absorption and increasing fat oxidation. Int. J. Obes. Relat. Metab. Disord..

[48] Ramachandra R., Shetty A.K., Salimath P.V. (2005). Quercetin alleviates activities of intestinal and renal disaccharidases in streptozotocin-induced diabetic rats. Mol. Nutr. Food Res..

[49] Shahkhalili Y., Finot P.A., Hurrell R., Fern E. (1990). Effects of foods rich in polyphenols on nitrogen excretion in rats. J. Nutr..

[50] Beck S.A., Tisdale M.J. (1989). Nitrogen excretion in cancer cachexia and its modification by a high fat diet in mice. Cancer Res..

[51] Tisdale M.J., Brennan R.A., Fearon K.C. (1987). Reduction of weight loss and tumour size in a cachexia model by a high fat diet. Br. J. Cancer.

[52] Padidar S., Farquharson A., Williams L., Kearney R., Arthur J., Drew J. (2012). High-fat diet alters gene expression in the liver and colon: Links to increased development of aberrant crypt foci. Dig. Dis. Sci..

[53] Barth S.W., Faehndrich C., Bub A., Watzl B., Will F., Dietrich H., Rechkemmer G., Briviba K. (2007). Cloudy apple juice is more effective than apple polyphenols and an apple juice derived cloud fraction in a rat model of colon carcinogenesis. J. Agric. Food Chem..

[54] Matsunaga K., Katayama M., Sakata K., Kuno T., Yoshida K., Yamada Y., Hirose Y., Yoshimi N., Mori H. (2002). Inhibitory effects of chlorogenic acid on azoxymethane-induced colon carcinogenesis in male F344 rats. Asian Pac. J. Cancer Prev..

[55] Fang M., Chen D., Yang C.S. (2007). Dietary polyphenols may affect dna methylation. J. Nutr..

[56] Xiao H., Hao X., Simi B., Ju J., Jiang H., Reddy B.S., Yang C.S. (2008). Green tea polyphenols inhibit colorectal aberrant crypt foci (ACF) formation and prevent oncogenic changes in dysplastic ACF in azoxymethane-treated F344 rats. Carcinogenesis.

[57] Hao X., Bose M., Lambert J.D., Ju J., Lu G., Lee M.-J., Park S., Husain A., Wang S., Sun Y. (2007). Inhibition of intestinal tumorigenesis in ApcMin/+ mice by green tea polyphenols (polyphenon e) and individual catechins. Nutr. Cancer.

[58] Tan C.W., Hirokawa Y., Gardiner B.S., Smith D.W., Burgess A.W. (2013). Colon cryptogenesis: Asymmetric budding. PLoS ONE.

[59] Oh S., Gwak J., Park S., Yang C.S. (2014). Green tea polyphenol EGCG suppresses Wnt/β-catenin signaling by promoting GSK-3β- and PP2A-independent β-catenin phosphorylation/degradation. BioFactors.

[60] Shimizu M., Deguchi A., Joe A.K., Mckoy J.F., Moriwaki H., Weinstein I.B. (2005). EGCG inhibits activation of HER3 and expression of cyclooxygenase-2 in human colon cancer cells. J. Exp. Ther. Oncol..

[61] Goldstein N.S., Armin M. (2001). Epidermal growth factor receptor immunohistochemical reactivity in patients with American Joint Committee on Cancer Stage IV colon adenocarcinoma: Implications for a standardized scoring system. Cancer.

[62] Kotronen A., Seppänen-Laakso T., Westerbacka J., Kiviluoto T., Arola J., Ruskeepää A.L., Yki-Järvinen H., Oresic M. (2010). Comparison of lipid and fatty acid composition of the liver, subcutaneous and intra-abdominal adipose tissue, and serum. Obesity.

[63] Wachira A.M., Sinclair L.A., Wilkinson R.G., Enser M., Wood J.D., Fisher A.V. (2002). Effects of dietary fat source and breed on the carcass composition, n−3 polyunsaturated fatty acid and conjugated linoleic acid content of sheep meat and adipose tissue. Br. J. Nutr..

[64] Green C.J., Hodson L. (2014). The influence of dietary fat on liver fat accumulation. Nutrients.

