# Supplementary material for: Azoxymethane-Induced Colorectal Cancer Mice Treated with a Polyphenol-Rich Apple Extract Show Less Neoplastic Lesions and Signs of Cachexia
Source: Foods. 2021 Apr 15;10(4):863. doi: 10.3390/foods10040863 (PMC8071383; doi:10.3390/foods10040863)
Supplement: Supplementary file 1 [file foods-10-00863-s001.zip › foods-1168955-supplementary.pdf]

Supplementary material

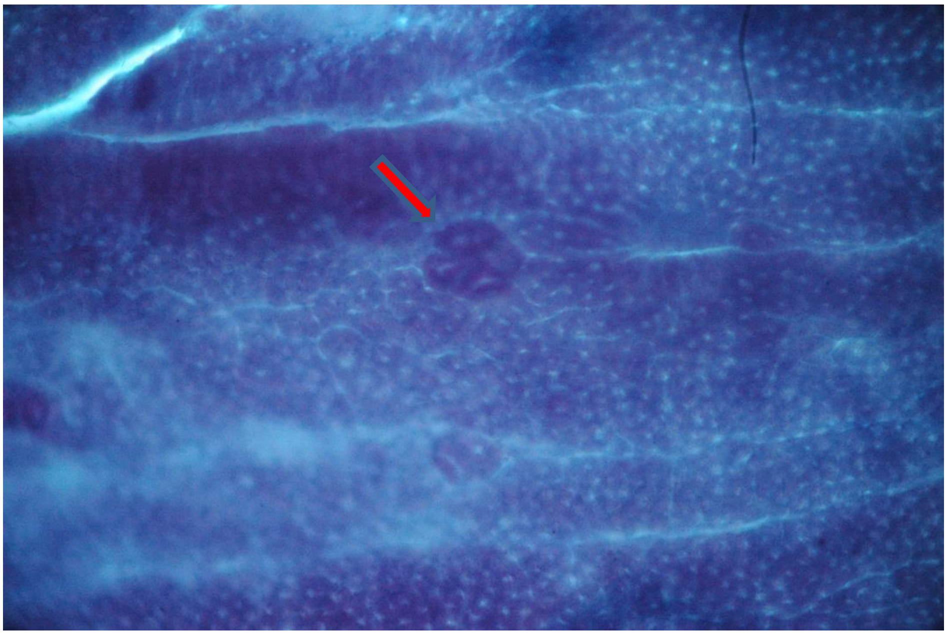

Figure S1. Topographical view of ACF. The colons were opened, stained with methylene blue solution and observed on a glass slide by transillumination in an optic microscope (Olympus). Increased size, bright blue staining and flat appearance hidden in the surrounding mucosa were used a criteria to identify ACF (arrow). Original magnification: 10×.
